# Distinguishing trans women in men who have sex with men populations and their health access in East Africa: A Tanzanian study

**DOI:** 10.4102/phcfm.v14i1.3428

**Published:** 2022-08-11

**Authors:** John Kashiha, Michael Ross, Nic Rider

**Affiliations:** 1 Community Health Education Services and Advocacy (CHESA), Dar es Salaam, Tanzania; 2 Department of Family Medicine, Faculty of Medicine, University of Minnesota, Minneapolis, Minnesota, United States of America

**Keywords:** trans women, transgender, Tanzania, Africa, stigma, prevalence, gender spectrum, healthcare

## Abstract

**Background:**

Few data are available on the presence and characteristics of transgender populations in sub-Saharan Africa (SSA), which makes the provision of health services for key populations difficult.

**Aim:**

This study aimed to ascertain the presence and characteristics of trans women in seven cities in Tanzania, East Africa.

**Setting:**

Tanzania, East Africa.

**Methods:**

Outreach to men who have sex with men (MSM) in seven large cities in Tanzania was carried out by non-governmental organisation (NGO) staff familiar with this community. Survey questions administered via interviews were used to identify participants who self-identify as trans. From the self-identification data, an estimate of the relative size of the trans women population in this sample was calculated.

**Results:**

In the sample of 300 participants, 17.0% of participants were identified as ‘transsexual or transgender’ (survey wording); 70.1% of these trans participants indicated that they identify themselves as a woman. Of those identifying themselves as transsexual or transgender, 43.1% reported living part- or full-time as a woman and eight (15.0%) reported hormone use. The highest percentage of hormone use (40.0%) was found in those living as a woman full-time. Notably, there was significant ignorance amongst the sample of the terms ‘transsexual and transgender’ or their explanation in Swahili, reported by interviewers.

**Conclusion:**

In this study, it is clear that trans women populations exist in Tanzania, with high levels of stigmatisation and threats to their lives. They should be included in health outreach and services to key populations. One in six self-identified as trans women, although the lack of knowledge of this concept in Swahili or English may have inaccurately represented numbers.

## Introduction

The literature on transgender and gender-diverse people in sub-Saharan Africa (SSA) is relatively sparse, partly because of the wide variation across cultures and traditions in the 46 countries that comprise SSA. It is also partly because the legal, religious, health and medical traditions that were introduced by colonial powers from the 19th century up to independence widely varied with regard to sexuality and have left their mark on subsequent legal and medical training.^1^ Whilst South Africa stands out by the breadth of its constitution with regard to recognition of human rights, gender and sexuality, significant gaps continue to exist between public and professional attitudes and the vision of the constitution. In other countries, legal, healthcare and public attitudes follow the whole range of responses to gender and sexual diversity from rejection to tolerance or recognition. It is therefore important to recognise that the situation in SSA may bear little coherence from country to country and culture to culture and that generalisability by geographic region is limited.

## Definitional issues

We will use the current terminology, trans woman, to refer to a person who identifies as a woman and whose sex assigned at birth was male, based on the World Health Organization’s (WHO) definition: ‘[*t*]ransgender is an umbrella term that describes a diverse group of people whose internal sense of gender is different than that which they were assigned at birth’.^2^ We also follow the suggestion of Klein^3^ that:

Even though [*trans and intersex*] people are often discussed as a single group, the complex intersectionality of for example class, religion, ethnicity, skin colour, income, and so on, places individuals at very different positions in life and lets them inhabit this ‘single’ category in very diverse ways. (p. 17)

Klein’s acknowledgement that there are a number of complex intersectionalities with trans identities is important to appreciate how research findings may differ depending on historical, geographical and cultural contexts.

## The African situation

Klein (p. 18) cited Donham^4^ as stating that ‘in the 1960s and 70s feminine urban black South African men were considered to be women (or people with variations of sex development) and their partners heterosexual men’. This alerts us to the fact that culture, race and time may be amongst the variables that determine whether classifications, imposed or adopted, of men who have sex with men (MSM) who may be feminine may have considerable variation, depending on cultural norms and medical classification, and more recently informed by Internet access. Time (during the apartheid era) and space (the apartheid terms were ‘coloured’ or ‘black’ townships – underdeveloped, racially segregated urban areas) along with particular terminology (the Afrikaans-originating term ‘moffie’, meaning effeminate gay man) all have specific South African roots.

We also know that some MSM (including in Africa) may consider themselves to be feminine or even use feminine terms for themselves, but actually identify as cis men, whereas there are others who don’t identify as men or who consider themselves at some intermediate point on the gender spectrum. The distinction between MSM and trans women, and the reference to trans women as ‘MSM’ in some studies in SSA, makes research focused on trans women specifically crucial to understand the separate political, health and social issues that trans women encounter. Their invisibility or misclassification as MSM makes it challenging to provide separate services tailored to trans women’s needs as a separate key population. One cannot assume that definitions and needs of trans women are identical in different countries or cultures in SSA. It is thus important to understand the history, culture and linguistic settings (including social and structural ones) of trans women or people on the gender spectrum in the specific context of any study.

## Historical issues in East Africa

Moen et al.,^1^ in a comprehensive review of same-sex practising men in Tanzania over the previous 150 years, noted that ‘the journey both begins and ends with terminology borrowed from overseas’. Whilst he observed that most of the terms which refer to MSM in Tanzania are based on sexual positioning as penetrating or receptive, reports from the British consul in Zanzibar, in the late 1850s, refer to ‘sodomites’ in Zanzibar as men who walked about dressed in female attire, with veils on their faces. Later reports in the early 1900s and 1990s refer to festival musical events which were danced by men in women’s clothing. Thus, people assigned male at birth who wore female clothing or exhibited mannerisms of women at that time were conflated with MSM. There is some evidence, cited by Moen et al.,^1^ that the conception of ‘homosexuality’ at that time was sometimes of men appearing as women. Regardless of how such behaviour might be viewed currently, it is clear that it occurred historically, and quite recently, it was (or is) viewed as being related to ‘homosexuality’. His observation that terminology borrowed from overseas (earlier, from Arabic and Persian) was being used should not only alert us to the problems of using Western diagnostic categories to label behaviours, including attire, which fall on the gender spectrum, but it also may include presentations which in Western culture might appear equivalent to transgender.

We need to be alert to cultural differences in terminology and tradition before imposing Western definitions and concepts regarding sexuality and gender. Moen et al.^5^ cited a survey amongst 500 same-sex practising men in Nairobi, Kenya (immediately to the north of Tanzania and with Kiswahili the most common language), in 2004 found that 46% of MSM referred to themselves as *gay*, whilst 23% called themselves *bisexual*, 16% *homosexual* and 12% *shoga*. However, he also noted that direct comparisons between Dar es Salaam and Nairobi may not necessarily be appropriate, because English is more widely spoken in Kenya than in Tanzania. Hersi,^6^ interviewing a transgender woman in Tanzania, reported that she was called *shoga, ssenge* and *Malaya* (homo, pervert, whore) and that most people conflate sexual orientation and gender identity, making no distinction between them. We cannot assume that *shoga* and related terms have any clear equivalence to Western categories.

## Recent African research

Recent studies have provided more information on transgender populations in the continent. Poteat et al.^7^ used respondent-driven sampling (RDS) of MSM in 14 sites in eight countries in SSA, including Burkina Faso, Côte d’Ivoire, Gambia (snowball sampling), Lesotho, Malawi, Senegal, Swaziland and Togo, between 2011 and 2016. Amongst the 4586 participants assigned male sex at birth, 20% identified as transgender or female, and the remainder identified as cisgender men. Transgender women were defined as participants who were assigned male at birth and self-identified as transgender, female or woman in the survey questionnaire. The data from a number of studies of MSM were combined. The largest proportion of transgender participants was from Côte d’Ivoire (33%), followed by Senegal (21%), Swaziland (13%), Burkina Faso (12%), Malawi (8%), Lesotho (8%), Togo (6%) and Gambia (< 1%). These wide differences in proportions may be because of the selection of seeds used for the RDS or differences in definition or language across cultures. Transgender women compared with cisgender men in the combined sample were more likely to have significantly higher levels of condomless receptive anal intercourse, higher levels of depression and higher experience of violence, sexual assault and stigmatisation by police.

Sandfort et al.^8^ assessed HIV incidence in transgender women in sites in South Africa, Malawi and Kenya. They found that HIV in transgender women was from 1.3/100 in Malawi to 14.4 person-years in South Africa. At baseline, 329 MSM and transgender women tested negative for HIV infection. Of this sample, 16.4% reported identifying as female or transgender. A few years earlier, Fearon et al.^9^ carried out an RDS study of MSM and trans feminine individuals regarding HIV testing and viral suppression in Johannesburg, and of their sample (*n* = 300), 77.7% identified as cisgender men, 14.7% as trans feminine and 7.0% as nonbinary individuals. Similarly, Rwema et al.^10^ also used RDS to study HIV prevalence and provision on healthcare services amongst transgender women and MSM (*n* = 736) in Kigali, Rwanda, and noted that 14.0% of their sample were transgender women.

It is not surprising that there is a lack of understanding of trans women as being distinct from MSM. In a qualitative study in KwaZulu-Natal, Luvuno et al.^11^ reported that healthcare workers (HCW) are uncertain about transgender patients, citing a lack of information and research on this key population, along with their own religious and cultural beliefs. Few studies in SSA have distinguished trans women from MSM populations or provided data that would help HCWs differentiate these key populations.

In Nairobi, Kenya, Smith et al.^12^ conducted an RDS study of 612 participants who have sex with men (86% were categorised as cisgender men, 11% as trans feminine, 1% as trans masculine and 2% as not identifying a gender). Trans feminine respondents were significantly more likely to be HIV-positive, more likely to report current symptoms of rectal STIs, more likely to report ≥ 4 male sex partners in the past three months, more likely to have condomless anal sex and receptive anal intercourse, more likely to have transactional sex and more likely to experience sexual assault. They concluded that the trans feminine population has a significantly greater risk of HIV and STIs compared with cisgender MSM. Effectively, transgender and gender diverse communities are a key population, and future research specifically focused on transgender and gender diverse communities is needed. Commenting on this, Chakrapani^13^ underscored the need for more data on transgender populations in Africa and elsewhere. This echoes the work of Kimani et al.^14^ in coastal Kenya, where in an HIV incidence cohort study, they found that 25% were exclusively MSM, and an additional 14% were transgender women. They convincingly argued that studies should assess if transgender women have been miscategorised as MSM in other studies in SSA.

Definitions and sampling in many studies of trans people in SSA have used differing definitions and sampling approaches. The dearth of knowledge in the area is because of many factors, such as those intersectionalities identified by Klein,^3^ stigma affecting sexual and gender diverse individuals, the need for sexual and gender diverse individuals to live ‘underground’ to escape threats and violence and the often subtle gender role and identity variations along these continua which make clear categorical distinctions difficult. Sample recruitment occurred through the gay community and networks, as both our previous community research and our key informants suggested that this was where trans women were most likely to interact.

Furthermore, there is a lack of awareness in healthcare and administrative areas that trans women are a key population with health needs that are different in many ways from those of MSM. This can include professionals not distinguishing trans women and gay and bisexual men (and confusing sexual orientation and gender identity). Thus, an investigation into the population and community access to trans women will allow planning and providing services to this key population.

## Methods

### Design

We carried out an interview-based study with a sample of 300 MSM, and the study was primarily on gender and sexual orientation-related stigma.^15^ Questions included gender role on a masculine–feminine continuum, identifying as a woman, living as a woman (including by wearing female attire part- or full-time and using hormone therapy, all considered options someone may choose to live as their gender) and exploring the presence of trans and gender diverse populations assigned male at birth. We further attempted to estimate their proportion as a key population within (and often confused with or miscategorised as) another key population, cisgender MSM.

### Setting

The study was carried out in seven of the major cities in Tanzania. Participants were interviewed in six of the 10 largest cities in Tanzania (i.e. Dar es Salaam, Mwanza, Arusha, Mbeya, Tanga, and Unguja, Zanzibar), plus Iringa, a city in the southern highlands. Census populations in 2012 were Dar es Salaam (4 364 541), Mwanza (706 543), Arusha (416 442), Mbeya (385 279), Tanga (273 332), Unguja, Zanzibar (223 033), and Iringa (151 345).^16^

### Sampling

Participants were recruited through peer educator links, social media, phone call appointments, the Community Health Education Services & Advocacy (CHESA) database, MSM groups and organisations working with and for MSM or gay and bisexual men, organisers of a community-based organisation (CBO) and snowball contacts. Administration of the interview survey was conducted at agreed-upon safe locations where the participant felt most comfortable and made the choice of place themselves, including bars, bus stations, garages, workplaces, accommodation, colleges or universities and in cars. Interviewing took place at participant-chosen locations (instead of at specific NGOs), given the political situation at the time of data collection, including condemnatory speech and incitement against MSM from community leaders and negative media exposure. Research took place from December 2017 to August 2018 (the period went longer than expected because of the anti-gay political and media issues). Interviews took up to about 30 min to complete but typically took about 15 min (the time available was open-ended).

### Data collection

Bilingual (in at least Kiswahili and English) research assistants trained community outreach workers and members of a CBO providing services to key populations (CHESA) to administer the interview-based questionnaire. Interviews were completed with 300 cisgender men and transsexual or transgender individuals over age 18 who reported having sex with men in the past five years. All survey administration took place in Kiswahili or English, as the participant preferred. The study was introduced as being about MSM, and transgender or transsexual identification was not mentioned in recruitment, thus not creating a participation bias. Interviewers asked questions, clarified responses and recorded interviewee information on the 21-item questionnaire, including both quantitative and open-ended responses, which was pretested with five members of the MSM community (including individuals who self-identify within the trans and gender diverse spectrum). At the time of the interview administration, which involved quantitative and open-ended questions, the interviewers explained the purpose of the research, and participants gave verbal informed consent. No incentive was provided.

One of the first three questions for participants was, ‘Do you consider yourself: gay or homosexual; bisexual; straight or heterosexual; transsexual or transgender; other (specify)…’ Participants were instructed to select one category. The participant’s response to questions was accepted without any additional questioning, unless the participant indicated that the question was unclear, or their answer was unclear. All interviewer training specifically indicated that transsexual or transgender people and people who considered themselves on the gender spectrum were part of the target population.

The questionnaire collected data on age; education level; sexual identity; sexual activity over the past five years; how masculine or feminine the participant thought of themselves (5-point Likert scale from ‘very feminine’ to ‘very masculine’); whether the participant had ever been the victim of physical violence or abuse, verbal abuse, discrimination or humiliation, sexual abuse or abuse from a healthcare worker (HCW) (Yes or No); whether the participant had ever visited an HCW because of a sexually or genitally related condition; whether the HCW was told (or found out) that the participant had sex with other men; and the types of abuse encountered in a healthcare setting. Depending on language, the two terms ‘transsexual’ and ‘transgender’ were used (in English), and the expression *mtu aliyebadilisha/wabadili jinsia* (‘someone who changed gender’) was used in Kiswahili. These terms and this expression were used together if there seemed to be any confusion. Based on our pilot work, the term ‘transsexual’ was the only English term that was understood in Tanzania to refer to what in Western societies is referred to as transgender, so we used those terms and a Kiswahili translation: 195 interviews were in Kiswahili, with only five being conducted in English. A copy of the questionnaire appears in the Appendix.

### Data analysis

Data were entered into Statistical Package for Social Sciences (SPSS) version 27 for analysis. For demographic data, means, SDs, medians and modes were calculated for linear data, and frequencies and percentages were calculated for categorical data. Comparisons between trans women and MSM were calculated for interval data by *t*-tests (separate variance estimates, two-tailed) and for ordinal data by χ^2^-tests (with Yates correction for discontinuity). Interviewer debriefing notes were also consulted for completeness of interpretation and to record any unanticipated issues, but they were not subject to any specific analysis.

### Total sample demographics

The parent study sample^15^ had a median age of 27 years, and the median age of first sex with a man was age 12 and with a woman was age 15. Forty-five percent reported that they self-identified as gay or homosexual, 32% bisexual, 6% heterosexual and 17% transsexual or transgender. Modal education was primary school (39%), and a quarter (26%) had completed some high school. Abuse based on one’s sexuality or gender role was reported as physical abuse by 41%, verbal abuse (63%), discrimination or humiliation (59%), sexual abuse (20%) and healthcare abuse (18%). A large majority (84%) had sought healthcare for a sexually related condition, and of those responding, only 24% indicated that they had disclosed or confirmed to the healthcare worker that they were MSM. Participants were not asked if they had confirmed they were transsexual or transgender to a healthcare worker. Interviews were carried out in six of the major cities in Tanzania, with a third (32%) coming from the major city, Dar es Salaam.

### Ethical considerations

Ethical clearance to conduct this study was obtained from the Institutional Review Board (IRB) of the University of Minnesota (ref. no. STUDY00001617). No details that might identify any participant were recorded. Quantitative data were entered into SPSS and checked against the original paper interview copy during data cleaning.

## Results

**FIGURE 1:**
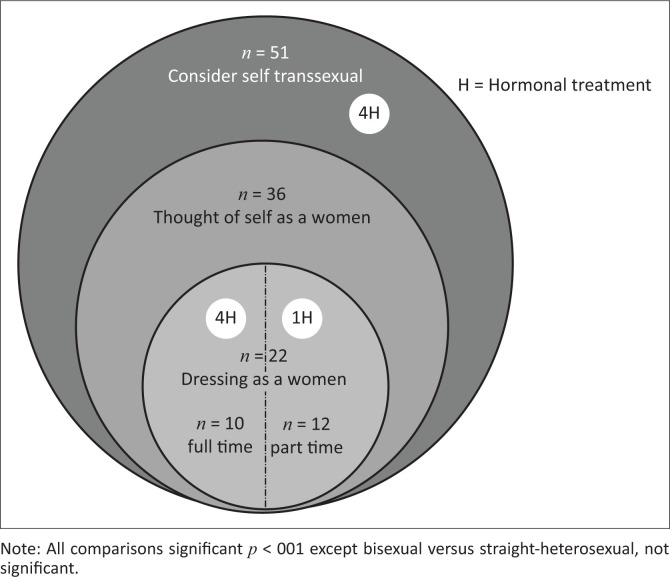
Breakdown of ‘transsexual and transgender’ sample.

Results are illustrated in Tables 1 and 2 and Figure 1. In Table 1, trans women reported that they were significantly younger at the age of first sex with a man and had fewer sexual partners in the past 12 months than the MSM in the sample. Trans women reported significantly higher experiences of physical, verbal and healthcare-related abuse than the MSM sample, but there were no significant differences in age, education level, sexual abuse or city of interview.

**TABLE 1a:** Comparison of Trans Women† compared with men who have sex with men participants.‡

Variable	Mean ± s.d.		*t*	*df*	*p*
	TW	MSM			
Age	28.94 ± 7.38	29.27 ± 7.22	−0.30	72.98	0.77
Age of first sex with a man	8.42 ± 4.57	12.85 ± 5.03	5.68	62.87	0.001
Number sexual partners in past 12 months	10.53 ± 7.16	8.38 ± 6.55	2.03	6.55	0.47

TW, Trans Women; MSM, men who have sex with men; *df*, degree of freedom.

† , Identify themselves as a woman, *n* = 51;

‡ , Not all n’s sum to 300 or percentages sum to 100 because of missing values.

**TABLE 1b:** Comparison of Trans Women† compared with men who have sex with men participants.‡

Variable	MSM (*n* = 249)	TW (*n* = 51)	χ^2^	*df*	*p*
**Education**					
None	3	1	9.15	5	0.33
Primary	95	21			
Some High School	70	11			
Completed High School	44	13			
Some tertiary	8	2			
University/College graduate	29	3			
**Abuse experiences**					
Physical abuse			15.41	5	0.33
Yes	88	34			
No	158	17			
**Verbal abuse**			18.53	1	0.0002
Yes	143	46			
No	102	5			
**Discrimination/humiliation**			3.21	1	0.07
Yes	133	44			
No	44	7			
**Sexual abuse**			0.24	1	0.62
Yes	51	9			
No	191	41			
**Healthcare abuse**			6.60	1	0.01
Yes	27	13			
No	211	36			
**City of interview**			3.75	6	0.71
Arusha	31	3			
Dar es Salaam	81	16			
Iringa	26	7			
Mbeya	38	8			
Mwanza	30	11			
Tanga	24	3			
Unguja/Zanzibar	19	3			

† , Identify themselves as a woman, *n* = 51;

‡ , Not all *n*’s sum to 300 or percentages sum to 100 because of missing values.

**TABLE 2:** Breakdown of ‘transsexual’ category.

Variable	*N*	%	Mean ± s.d.	Median
**Consider oneself transsexual?**				
Yes	51	17.0	-	-
No	249	83.0	-	-
How old when thought of oneself as a woman?	36	-	12.42 ± 3.43	12
How old when started dressing as a woman?	22	-	14.00 ± 2.92	14
Hormone treatment	9	17.7†	-	-

s.d., standard deviation.

† , Percentage of those saying are ‘transsexual’.

Table 2 indicates that of the 17.0% considering themselves ‘transsexual or transgender’ (the words used in the interview), about two-thirds (70.1%) *thought* of themselves as women, and of those considering themselves transsexual or transgender, 43.1% reported *living* part- or full-time as women. Eight of those indicating that they were transsexual or transgender (15.0%) reported hormone use. The highest percentage of hormone use (40.0%) was found in those living as women full-time (Figure 1).

Debriefing of interviewers was carried out by the first author (J.K.). In his notes, the interviewers reported (accounts are from interviewers and in their words, describing spontaneous verbal comments made by interviewees) that many (about a quarter or less) participants had to have the interviewer explain the terms transsexual, transgender and/or *mtu aliyebadilisha/wabadili jinsia* to them and were not familiar with the term or the concept.

From this hearsay information, fear of discrimination and rejection was a major reason that some transsexual or transgender participants did not wear women’s clothes, as fully dressing as a woman would make their life much more dangerous compared with hiding it. From the debriefing notes:

‘Some of the participants were actually telling us [*interviewers*] off the cuff that they make sure that they have even just one piece of women on her body, or accessories, and that makes her feel confidence and a natural woman.’ (Interviewer, no date)

So they may wear women’s underwear or carry lipstick in their bags or anything else that helps them feel like women. But publicly, they cannot show their identities or dress like women. In debriefing notes, an interviewer explained:

‘One of the participants told us that “I bought high heeled shoes and kept them in my wardrobe, and every day I came home I made sure that I touched them or was wearing them in my room. Sometimes I slept with them on till morning and I took them off.”’ (Interviewer, no date)

An interviewer also wrote that another participant ‘showed the wedding gown she bought five years back; she told us that she “usually wears it whenever she feels down or when she hears negativity from society”’. The interviewer further described that the participant:

‘Just waited for that moment where she could be free to choose who to love, get married and live as husband and wife of someone, and if that won’t happen when she is alive, she asked friends to put that dress on her body during her funeral as to fulfil her dreams.’ (Interviewer, no date)

## Discussion

Whilst there are data from other studies in SSA that report on trans women or gender non-binary populations within MSM sampling, few explicitly distinguish them, and the definitions are not always similar. Historical studies confirm the existence of trans women or nonbinary people prior to European colonisation. This study investigates the situation in a large MSM sample in seven Tanzanian cities and identifies the presence of trans women within the greater MSM population.

These data are amongst the few studies in East Africa to explore the existence of transsexual or transgender women within an MSM population. Our interviewers noted that many participants did not know what ‘transsexual or transgender’ meant, and it had to be explained to them: the fact that it was possible to change one’s sex or gender was unknown to a significant number of the sample. This suggests that even within MSM communities, trans women are a hidden population. Several interviewers also commented in their field notes that they thought that some of the samples believed that ‘thinking of themselves as a woman’ referred to sexual positioning as a ‘bottom’. This confusion and lack of a word to describe it undoubtedly raised the number of responses identifying as transsexual or transgender. However, this was not a random sample, and the figures here cannot be used to accurately estimate the number of transsexual, transgender or trans feminine people in this population. Nevertheless, it does document the existence of a number of trans and gender diverse people assigned male at birth in Tanzania. Nearly half of those who identified themselves as transsexual or transgender acknowledged that they had part- or full-time dressed or lived as women, and a small proportion reported taking hormones (of unknown provenance but possibly black market). There are no surgical interventions known for transsexual people in Tanzania, and no participant reported them.

As previously mentioned, the concept or vocabulary of transsexual or transgender was not consistently known to the MSM community we surveyed in Tanzanian cities: this was the major challenge we encountered during data collection. Interviewers took time to explain this concept so as to help people understand and to categorise themselves and be able to respond to the questions. However, despite these explanations, we could not be sure if respondents thought this was referring to culturally perceived gender-role appropriate mannerisms or sexual positioning, so their responses may be more likely based on the roles they played in society and/or sexually. Thus, we used a combination of markers to consider the size of this key population.

As the interviewer debriefing notes showed, there was a general recognition amongst those who did identify as ‘transsexual or transgender’ that to wear women’s clothing was to risk their lives or livelihoods. Thus, it is not surprising that there was a lack of recognition in this population of transsexual or transgender women assigned male at birth. From Figure 1, that may be because one in six of those who consider themselves transsexual or transgender live full-time or part-time as women and would most likely very carefully protect their identity given the probability of negative, indeed fatal, consequences if there was gossip. Hersi^6^ gave a case history of a transsexual woman in Dar es Salaam who had her identity publicly exposed and who had to immediately flee to a neighbouring country rather than be killed.

Nevertheless, there was a core of participants who we believe did understand the term and justify the description of transgender or transsexual (Figure 1). Despite the cultural, religious and legal discrimination and risk associated with being sexual and gender minorities in Tanzania, some participants still reported living as women, nine participants reported taking hormones and of these, five dressed full-time or part-time as women. As Figure 1 illustrates, as we increase the possible combination of criteria to identify a transsexual or transgender population, numbers decrease.

We believe that we made assumptions about the knowledge level of some participants: the concepts of transsexual or transgender people seem not to be universally recognised. On the contrary, in the positive responses we did get, we can identify a clear gender continuum inclusive of trans and gender diverse people assigned male at birth, despite the lack of use of terminologies such as transgender or transsexual.

In our opinion, using the terms transsexual, transgender or their Kiswahili explanation underlies an issue that may occur in many countries in SSA: the use of a Western term where there is no understanding of that term or no cultural equivalent to that status. Lorway,^17^ in a rich ethnographic study of ‘Rainbow youth’ in Windhoek, Namibia, illustrates how sexual or gender categories used by transnational activists may be adopted without a consideration of their appropriateness in the political, economic or social context of SSA. Lorway noted that this can lead to a refiguring of identity categories and subjectivities as youth adopt Western categorisations. In Tanzanian cities, gender minorities, when faced by a term or phrase that does not have an equivalent, may or may not define their own identity as fitting a Western label.

Our data indicate that 17.0% of an MSM sample identified as transsexual or trans women, compared with 20.0% in Poteat’s^7^ data (showing a range across eight countries from 33.0% to 1.0%), 16.4% in Sandfort et al.’s^8^ South African data, 14.7% in Fearon et al.’s^9^ Johannesburg data, 14.0% in Rwema et al.’s^10^ Rwanda data, 11.0% trans feminine and 2.0% no gender in Smith et al.’s^12^ data in Nairobi; and in Kimani’s^14^ HIV incidence cohort data, 25.0% were MSM and 14.0% were transgender. Whilst these data are based on very different sampling methods and to a lesser extent definitions, the data suggest that a proportion of somewhere between 10.0% and 20.0% of MSM samples in SSA being transsexual, transgender or nonbinary would be a reasonable estimate. However, the lack of random sampling makes it difficult to estimate with greater precision and confidence. Nevertheless, it is clear that transsexual or transgender individuals assigned male at birth make up a significant key subpopulation of the so-called MSM samples. Given the variation across Hersi’s^6^ 8-country samples, however, it may be that definition in terms of local culture or language (especially terms or phrases used from other languages), along with sampling frames or RDS seed selection, has a large impact on the population accessed.

On the contrary, our data also identify a group who identify themselves as ‘transsexual or transgender’, female and women; they may dress or live (fully or partially) as women in the face of discrimination and high personal risk and use hormone therapy where available (from whatever source). From our data and interviewer debriefing comments, it appears that there is indeed a small group who in a Western context would be considered ‘transsexual or transgender’ and were assigned male at birth. Using the criterion of the WHO,^2^ ‘[*t*]ransgender is an umbrella term that describes a diverse group of people whose internal sense of gender is different than that which they were assigned at birth’, we identified three possible indicators (identifying as a woman; living as a woman, including by wearing female clothing part- or full-time; and using hormone therapy) that constitute about 7% of the MSM sample in this study. A further significant proportion appears to identify themselves as being on a gender continuum. We believe that a wider range of indicators, including sex assigned at birth, gender role identity, gender identity (as a woman, man or nonbinary), gender of sexual partners, living to any extent publicly or privately as a woman or use of hormone treatment, can provide helpful information of these key populations, as opposed to simply being defined by researchers as ‘MSM’.^14^

Abuse based on sexuality or gender role was reported as being physical abuse by nearly half of all participants, verbal abuse as nearly two-thirds, discrimination or humiliation by half, sexual abuse by one in five and healthcare abuse by one in six; this was significantly higher for trans women for physical, verbal and healthcare-related abuse. A large majority (eight out of 10) had sought healthcare for a sexually related condition, so it is possible that some participants may face a difficult choice between getting medical treatment and being abused. In our earlier work on this study,^16^ it was shown that healthcare discrimination and abuse were significantly associated with the reported femininity of the individual. That is, the more feminine, the more healthcare abuse. It is clear that there is significant stigmatisation for trans women and nonbinary people assigned male at birth. These data echo the study of Mujugira et al.^18^ in Uganda, which found that multiple levels and sources of stigma in trans men impeded access to healthcare services. The need for trans-friendly care noted in that study was mostly motivated by stigma avoidance in public facilities.

### Limitations

This study has several significant limitations. These include a small (by international standards) sample of people assigned male at birth and who have had sex with a man in the past five years, which in previous research contexts may have been described as ‘MSM’ and confined to the larger cities in Tanzania. We cannot, given our sampling and methodology, estimate a population figure for trans women and gender diverse people assigned male at birth. It became apparent that there was a substantial proportion of the sample who were not familiar with the term ‘transsexual or transgender’ and could have misunderstood its meaning. This could have underestimated our assessment. The term ‘nonbinary’ was not familiar to the interview designers, and no participants noted it in the ‘other’ self-identification category. Nevertheless, it is possible that the term ‘transsexual’ covered people who might define themselves as ‘nonbinary’ in the West if they were familiar with the term. Nor is there good agreement on classification criteria for trans women, particularly in the social, political, cultural and linguistic context of East Africa; other researchers may prefer different combinations of criteria. This will no doubt be a matter of continuing investigation across SSA.

## Conclusion

It is clear that there are trans and gender diverse people assigned male at birth in Tanzania who identify as and report that they live as women, as well as a larger number who because of the risks cannot safely live as women but may identify as such. Without doubt, there are identifiable ‘transsexual and transgender’ populations in Tanzania, as the terms are used in North America and Western Europe; they need to be identified as a key population separate from MSM, in health and healthcare education. This is especially so given the consistent evidence from African research noted that these key populations have been conflated. Importantly, there are recent South African gender-affirming standards of care, including for transgender or transsexual key populations, which are available and applicable to SSA healthcare settings.^19^ Whilst this study identifies the presence of trans women in urban areas in Tanzania, it is also apparent that this is a field in which rich histories of individuals and qualitative methodologies need to be emphasised to fully understand trans people assigned male at birth, in their personal, family, cultural, political, legal and structural context. Future research should focus on this.

## References

[1] Moen K, Aggleton P, Leshabari MT, Middelthon, A-L. Same-sex practicing men in Tanzania from 1860 to 2010. Arch Sex Behav. 2014;43(6):1065–1082. 10.1007/s10508-014-0286-2 24752788

[2] World Health Organization. International classification of diseases for mortality and morbidity statistics (11th rev.) [homepage on the Internet]. 2018 [cited 2021 Sep 29]. Available from: https://icd.who.int/browse11/l-m/en

[3] Klein T. Intersex and transgender activism in South Africa. Liminalis J Sex Gender Emanc Resist. 2009;3:15–41.

[4] Donham DL. Freeing South Africa: The ‘modernization’ of male-male sexuality in Soweto. In: Inda JX, Rosaldo R, editors. The anthropology of globalization: A reader. Oxford: Blackwell, 2002; p. 410–427.

[5] Moen K, Aggleton P, Leshabari MT, Middelthon AL. Gays, guys, and mchicha mwiba: Same-sex relations and subjectivities in Dar es Salaam. J Homosex. 2014;61(4): 511–539. 10.1080/00918369.2014.865457 24313863

[6] Hersi A. Tanzania: One transgender woman’s pain amid fears of arrest [homepage on the Internet]. 2018 [cited 2020 Mar 12]. Available from: https://www.amnesty.org/en/latest/campaigns/2018/11/tanzania-one-transgender-womans-pain-amid-fears-of-arrest/

[7] Poteat T, Ackerman B, Diouf D, et al. HIV prevalence and behavioral and psychosocial factors among transgender women and cisgender men who have sex with men in 8 African countries: A cross-sectional analysis. PLoS Med. 2017;14(11):e1002422. 10.1371/journal.pmed.1002422 29112689 PMC5675306

[8] Sandfort TGM, Dominguez K, Kayange N, et al. HIV testing and the HIV care continuum among sub-Saharan African men who have sex with men and transgender women screened for participation in HPTN 075. PLoS One. 2019;14(5):e0217501. 10.1371/journal.pone.0217501 31150447 PMC6544251

[9] Fearon E, Tenza S, Mokoena C, et al. HIV testing, care and viral suppression among men who have sex with men and transgender individuals in Johannesburg, South Africa. PLoS One. 2020;15(6):e0234384. 10.1371/journal.pone.0234384 32555703 PMC7299351

[10] Twahirwa Rwema JO, Lyons CE, Herbst S, et al. HIV infection and engagement in HIV care cascade among men who have sex with men and transgender women in Kigali, Rwanda: A cross-sectional study. J Int AIDS Soc. 2020;23(S6):e25604. 10.1002/jia2.25604 33000912 PMC7527755

[11] Luvuno ZPB, Ncama B, Mchunu G. Transgender population’s experiences with regard to accessing reproductive health care in Kwazulu-Natal, South Africa: A qualitative study. Afr J Prim Health Care Fam Med. 2019;11(1):1–9. 10.4102/phcfm.v11i1.1933 PMC6676963 31296016

[12] Smith AD, Kimani J, Kabuti R, Weatherburn P, Fearon W, Bourne A. HIV burden and correlates of infection among transfeminine people and cisgender men who have sex with men in Nairobi, Kenya: An observational study. Lancet HIV. 2021;8(5):E274–E283. 10.1016/S2352-3018(20)30310-6 33631101

[13] Chakrapani V. Need for transgender-specific data from Africa and elsewhere. Lancet HIV. 2021;8(5):E249–E250. 10.1016/S2352-3018(20)30344-1 33631100 PMC7611171

[14] Kimani M, Van Der Elst EM, Chiro O, et al. PrEP interest and HIV-1 incidence among MSM and transgender women in coastal Kenya. J Int AIDS Soc. 2019;22(6):e25323. 10.1002/jia2.25323 31194291 PMC6563853

[15] Ross MW, Kashiha J, Mgopa LR. Stigmatization of men who have sex with men in health care settings in East Africa is based more on perceived gender role-inappropriate mannerisms than having sex with men. Glob Health Action. 2020;13(1):1816526. 10.1080/16549716.2020.1816526 32985387 PMC7534303

[16] Wikipedia. List of cities in Tanzania [homepage on the Internet]. No date [cited 2021 Sep 29]. Available from: https://en.wikipedia.org/wiki/List_of_cities_in_Tanzania

[17] Lorway R. Namibia’s rainbow project. Gay rights in an African nation. Bloomington, IN: Indiana University Press; 2015.

[18] Mujugira A, Kasiita V, Bagaya M, et al. ‘You are not a man’: A multi-method study of trans stigma and risk of HIV and sexually transmitted infections among trans men in Uganda. J Int AIDS Soc. 2021;24(12):e25860. 10.1002/jia2.25860 34965322 PMC8716065

[19] Tomson A, McLachlan C, Wattrus C, et al. Southern African HIV clinicians society gender-affirming healthcare guideline for South Africa. South Afr J HIV Med. 2019;22(1):1299. 10.4102/sajhivmed.v22i1.1299 PMC8517808 34691772

